# A Longitudinal Analysis of SF-36 scores Within the Candrive Cohort: An Example of Survivor Bias

**DOI:** 10.1093/geroni/igaa057.1702

**Published:** 2020-12-16

**Authors:** Michel Bedard, Hillary Maxwell, Isabelle Gelinas, Shawn Marshall, Gary Naglie, Michelle Porter, Holly Tuokko, Brenda Vrkljan

**Affiliations:** 1 Lakehead University, Thunder Bay, Ontario, Canada; 2 McGill University, Montreal, Quebec, Canada; 3 University of Ottawa, Ottawa, Ontario, Canada; 4 University of Toronto, Toronto, Ontario, Canada; 5 University of Manitoba, Winnipeg, Manitoba, Canada; 6 University of Victoria, Victoria, British Columbia, Canada; 7 McMaster University, Hamilton, Ontario, Canada

## Abstract

A bias inherent to prospective studies is focusing only on individuals who remain in the study; these individuals may differ from those who leave early. To examine this issue, we analyzed SF-36 scores by completion status for individuals enrolled in the seven-year Candrive cohort. The SF-36 provides a self-reported evaluation of health and well-being along two subscales, the Physical Component Summary (PCS) and the Mental Component Summary (MCS). Of 928 participants in the cohort, 887 had at least two consecutive years of data starting at baseline (age=76.17, SD=4.81; 61.9% male). A total of 142 participants had 7 years of data. Study discontinuation (due to withdrawal, driving cessation, or death) happened least in early years, and peaked after 6 years (n=235). When analyzed according to completion status, patterns of change in SF-36 scores varied. For example, participants with 7 years of data had mean PCS scores ranging from 51.41 (SD=7.92) at baseline to 46.93 (SD=9.46) at year 7, a change of 0.75 points per year. For those with only two years of data, scores were lower and dropped from 45.82 (SD=9.98) to 43.59 (SD=10.90), a change of 2.23 points over a single year (p<.001). Differences are also evident for other groups. While the results indicate relative stability of SF-36 scores among participants who remained in the study, participants who dropped out reported greater deterioration in scores. These results highlight important differences between participants based on completion status.

